# Interactions of the Receptor Binding Domain of SARS-CoV-2 Variants with hACE2: Insights from Molecular Docking Analysis and Molecular Dynamic Simulation

**DOI:** 10.3390/biology10090880

**Published:** 2021-09-07

**Authors:** Ismail Celik, Rohitash Yadav, Zekeriya Duzgun, Sarah Albogami, Ahmed M. El-Shehawi, Fatimawali, Rinaldi Idroes, Trina Ekawati Tallei, Talha Bin Emran

**Affiliations:** 1 Department of Pharmaceutical Chemistry, Faculty of Pharmacy, Erciyes University, Kayseri 38039, Turkey; 2 Department of Pharmacology, All India Institute of Medical Sciences, Rishikesh 249203, India; rohitashyadav1@gmail.com; 3 Department of Medical Biology, Faculty of Medicine, Giresun University, Giresun 28100, Turkey; zekeriya.duzgun@giresun.edu.tr; 4 Department of Biotechnology, College of Science, Taif University, P.O. Box 11099, Taif 21944, Saudi Arabia; dr.sarah@tu.edu.sa (S.A.); elshehawi@hotmail.com (A.M.E.-S.); 5 Pharmacy Study Program, Faculty of Mathematics and Natural Sciences, Sam Ratulangi University, Manado 95115, Indonesia; fatimawali@unsrat.ac.id; 6 The University Center of Excellence for Biotechnology and Conservation of Wallacea, Sam Ratulangi University, Manado 95115, Indonesia; trina_tallei@unsrat.ac.id; 7 Department of Pharmacy, Faculty of Mathematics and Natural Sciences, Universitas Syiah Kuala, Kopelma Darussalam, Banda Aceh 23111, Indonesia; rinaldi.idroes@unsyiah.ac.id; 8 Department of Biology, Faculty of Mathematics and Natural Sciences, Sam Ratulangi University, Manado 95115, Indonesia; 9 Department of Pharmacy, BGC Trust University Bangladesh, Chittagong 4381, Bangladesh

**Keywords:** SARS-CoV-2, COVID-19, variant of concern, coronavirus-2, alpha variant, beta variant, gamma variant, delta variant

## Abstract

**Simple Summary:**

Since the onset of the COVID-19 pandemic in late 2019, SARS-CoV-2 has evolved via genetic changes, resulting in numerous variants of concern (VOCs) and interest (VOIs). Using protein-protein docking and dynamics simulation, we examined the interactions of five SARS-CoV-2 variations’ receptor-binding domains with the human angiotensin-converting enzyme 2 (hACE2) receptor in host cells. A comparison of protein-protein docking and dynamics simulations showed that these point mutations significantly altered the structural behavior of the spike (S) protein, affecting RBD binding to hACE2 at the respective sites. Further research is needed to determine whether these changes affect drug–S protein binding and its potential therapeutic impact.

**Abstract:**

Since the beginning of the coronavirus 19 (COVID-19) pandemic in late 2019, severe acute respiratory syndrome coronavirus 2 (SARS-CoV-2) has been evolving through the acquisition of genomic mutations, leading to the emergence of multiple variants of concern (VOCs) and variants of interest (VOIs). Currently, four VOCs (Alpha, Beta, Delta, and Gamma) and seven VOIs (Epsilon, Zeta, Eta, Theta, Iota, Kappa, and Lambda) of SARS-CoV-2 have been identified in worldwide circulation. Here, we investigated the interactions of the receptor-binding domain (RBD) of five SARS-CoV-2 variants with the human angiotensin-converting enzyme 2 (hACE2) receptor in host cells, to determine the extent of molecular divergence and the impact of mutation, using protein-protein docking and dynamics simulation approaches. Along with the wild-type (WT) SARS-CoV-2, this study included the Brazilian (BR/lineage P.1/Gamma), Indian (IN/lineage B.1.617/Delta), South African (SA/lineage B.1.351/Beta), United Kingdom (UK/lineage B.1.1.7/Alpha), and United States (US/lineage B.1.429/Epsilon) variants. The protein-protein docking and dynamics simulation studies revealed that these point mutations considerably affected the structural behavior of the spike (S) protein compared to the WT, which also affected the binding of RBD with hACE2 at the respective sites. Additional experimental studies are required to determine whether these effects have an influence on drug–S protein binding and its potential therapeutic effect.

## 1. Introduction

Severe acute respiratory syndrome coronavirus 2 (SARS-CoV-2), which causes coronavirus disease 2019 (COVID-19), has had a major impact on human health and socio-economic status globally [1,2]. SARS-CoV-2 is a single-stranded positive-sense RNA (+ssRNA) with a 29.9 kb genomic length and contains two large ORFs (ORF1a and ORF1b) which encode for 16 non-structural protein (nsp1-16) and structural protein encoding genes [3,4]. Morphologically, the virus consists of four structural proteins, namely spike (S), envelope (E), membrane (M), and nucleocapsid (N), among which the former three are integral membrane proteins and the latter remains complexed with its RNA genome. In addition, it comprises 9 or 10 accessory proteins [5,6,7].

At the genomic level, the region downstream of the 5′-untranslated region (UTR), which encompasses ORF1a and ORF1b, accounts for 67% of the total genome, and encodes viral replicase and protease. The remaining genomic region preceding the 3′-UTR possesses four ORFs that encode S, E, M, and N structural proteins, as well as 9 or 10 interspersed ORFs that correspond to accessory proteins [8,9,10].

**Figure 1:**
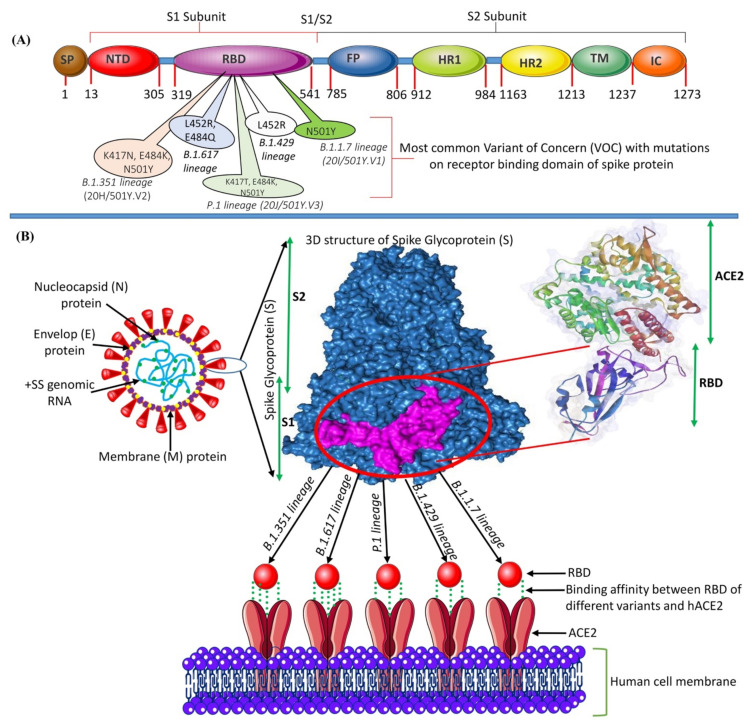
(**A**) The genomic structure of the S glycoprotein of SARS-CoV-2 showing the position of each gene and the impactful mutations in the RBD. (**B**) Structure of SARS-CoV-2 and the binding mode between the RBD of the S protein with the VOCs of SARS-CoV-2 and the host-cell receptor hACE2.

The spike (S) glycoprotein is a critical component of viral infection. It adheres to the host cell’s surface receptor, human angiotensin-converting enzyme 2 (hACE2), allowing viral cellular entry via endosome formation and/or plasma-membrane fusion [11,12]. The S protein (1273 amino-acid residues) exists in a trimeric prefusion form, which comprises an amino (N)-terminal signal peptide (SP) (residues 1–13), the S1 subunit (residues 14-685), and the S2 subunit (residues 686-1273) (Figure 1). It is thought that the host furin protease is responsible for the cleavage of the S protein into its S1 and S2 subunits [13]. The S1 subunit contains an N-terminal domain (residues 14-305) and a receptor-binding domain (RBD; residues 319-541). The S2 subunit contains a fusion peptide (FP) (residues 788–806), heptapeptide repeat sequence 1 (HR1) (residues 912-84), HR2 (residues 1163–1213), a transmembrane (TM) domain (1213–1237 residues), and a C-terminal cytoplasmic domain (residues 1237–1273). S1 and S2 are responsible for binding with the host-cell receptor and membrane fusion, respectively [14,15]. After entering the cell, the virus releases its genomic RNA into the cytoplasm. Both the 5′ ORF1a and ORF1b are immediately translated by host-cell ribosomes, forming precursor polypeptides that are referred to as pp1a and pp1ab. These then undergo autoproteolysis, forming 16 enzymatic nsps, which are assembled into a three-dimensional (3D) supramolecular enzymatic complex known as RNA-dependent RNA polymerase (RdRp). RdRp binds to the +ssRNA genome, forming a replication–transcription complex (RTC), which mediates these processes. The RTC activity results in the synthesis of sub-genomic mRNAs, whose translation produces a multitude of structural and accessory proteins [16,17].

**Figure 2:**
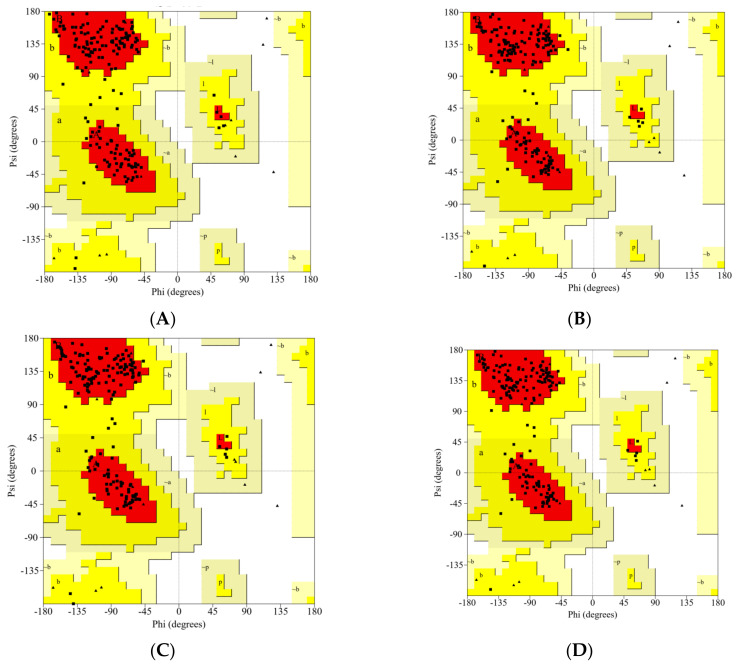

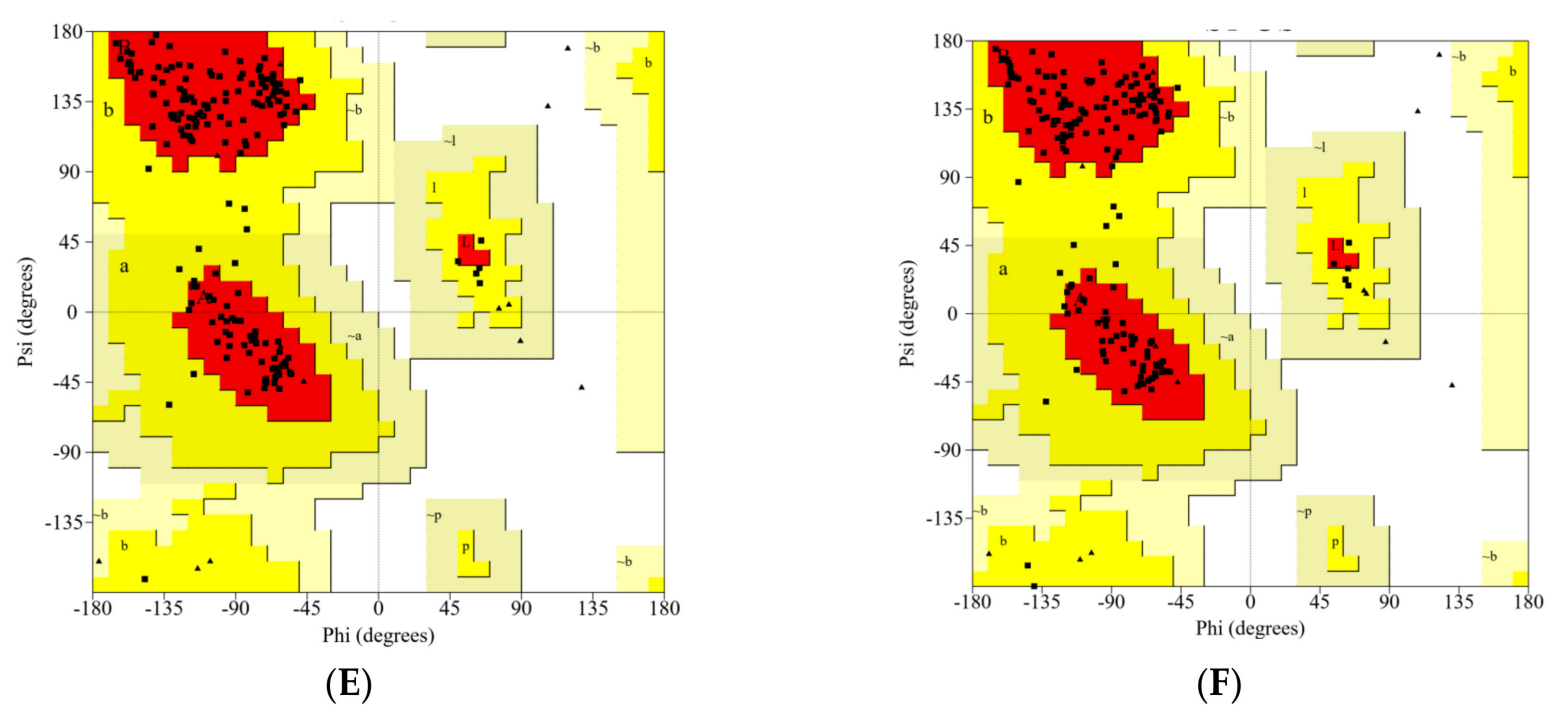
The Ramachandran plot analysis of the predicted models for the RBD variants: (**A**) WT, (**B**) Gamma; (**C**) Delta; (**D**) Beta; (**E**) Alpha; (**F**) Epsilon.

Since the beginning of the COVID-19 pandemic in late 2019, SARS-CoV-2 has been evolving through the acquisition of genomic mutations, leading to the emergence of multiple specific variants of concern (VOCs) and variants of interest (VOIs) (Figure 2). More recently, several VOIs and VOCs with the potential for increased transmissibility and virulence have been identified, which may enhance disease severity, as well as show resistance to the prevailing vaccination program worldwide [18,19,20,21]. The US government’s SARS-CoV-2 Interagency Group (SIG) and the European Centre for Disease Prevention and Control (ECDC) (https://www.ecdc.europa.eu/en/covid-19/variants-concern (accessed on 28 August 2021)) regularly evaluate new evidence on variants discovered through epidemic intelligence, rules-based genomic variant screening, or other scientific sources on a regular basis. They classified SARS-CoV-2 variants into three categories: variants of interest (VOI), variants of concern (VOC), and variants of high consequence (VOHC). They defined VOI as a genetic variant associated with altered receptor binding, reduced neutralization by previous infection or vaccination antibodies, reduced treatment efficacy, potential diagnostic impact, or predicted increase in transmissibility or disease severity. This includes Eta (B.1.525), Theta (P.3), Kappa (B.1.617.1), n/a (B.1.620), n/a (B.1.621), Iota (B.1.526), n/a (B.1.617.3), and Lambda (C.37). VOC is a variant with evidence of increased transmissibility, more severe disease (e.g., more hospitalizations or deaths), a significant reduction in neutralization by antibodies produced during previous infection or vaccination, and reduced treatment effectiveness. This includes Alpha (B.1.1.7), Beta (B.1.351, B.1.351.2, B.1.351.3), Delta (B.1.617.2, AY.1, AY.2, AY.3, AY.4, AY.5, AY.6, AY.7, AY.8, AY.9, AY.10, AY.11, AY.12), and Gamma (P.1, P.1.1, P.1.2). Meanwhile, VOHC is a variant of high consequence for which there is clear evidence that preventive or medical countermeasures (MCMs) are significantly less effective than previously circulating variants.

The global scientific community is rigorously following the three most rampantly spreading SARS-CoV-2 VOCs identified in the United Kingdom, South Africa, and Brazil [22,23,24]. The first SARS-CoV-2 VOC, B.1.1.7 (also known as 20I/501Y.V1, VOC202012/01, and the UK variant) was identified in the United Kingdom in December 2020 (Kirby, 2021). Within a few months, this variant became prevalent in the United Kingdom and has since expanded to 114 other nations worldwide [25,26]. This variant has around 17 mutations in the S protein alone, including a crucial nonsynonymous mutation (N501Y) at position 501 in the RBD in which asparagine (N) has been replaced with tyrosine [27].

Subsequently, another variant, B.1.351 (also known as 20C/501Y.V2 or the SA variant) was identified in South Africa with 21 mutations, including E484K, N501Y, and K417N in the S protein [28]. These three S-protein mutations involve glutamic acid (E), asparagine (N), and lysine (K) at positions 484, 501, and 417, being replaced by lysine (K), tyrosine (Y), and asparagine (N), respectively.

In January 2021, the variant P.1 lineage (also known as 20J/501Y.V3 or the BR variant), was first reported in four individuals travelling from Brazil to Japan. VOCs with 17 amino-acid changes, including N501Y, E484K, and K417N in the S protein, and ORF1b deletion, were identified in this variant [16,29]. COVID-19 variants have now been given non-stigmatizing Greek names by the World Health Organization (WHO). B.1.1.7, B.1.351, P.1, and B.1.617.2 lineages have been designated as the Alpha, Beta, Gamma, and Delta variants, respectively [30].

Mutations in the RBD can help to enable strong affinity and binding capacity to hACE2, leading to higher transmissibility [28,31,32]. Furthermore, these mutations may result in a reduction in antibody neutralization [33,34]. This could have a major impact on the efficacy of existing vaccines. This has the potential to have a significant impact on the efficacy of the existing vaccines [17,35]. As a result, global monitoring of the continuing genomic changes in SARS-CoV-2 is critical for identifying areas linked with drug resistance and vaccine evasion in order to create successful antiviral medicines. Several drug-repositioning studies and compound evaluations have been conducted to discover novel antiviral medicines against SARS-CoV-2 utilizing experimental and theoretical/computational methodologies [36,37,38].

Furthermore, genetic differences in the active/binding site region of molecular targets can have a significant impact on the binding mechanism and affinity of the lead compounds. Consequently, this will be of relevance to the efficacy of previously investigated promising candidates. The current study modelled the structure of the S protein of newly found variants and examined novel antiviral medications, utilizing a variety of computational drug design methodologies, with the aim of identifying a viable treatment for COVID-19.

## 2. Results and Discussion

The SARS-CoV-2 that causes COVID-19 continues to mutate. The majority of variants created through alterations in amino acids in the RBD have been found to be less infectious [39], but certain variations investigated have been resistant to some neutralizing antibodies [40]. A mutation that occurred outside the RBD region (D614G) was reported to be more infectious, although there was no evidence that this variant was resistant to neutralizing antibodies [39]. Mutations that occur in the RBD region are likely to have an impact on the attachment of the virus to hACE2 [41]. Therefore, the present study focused on analyzing the interaction between several RBD variants with hACE2 through molecular docking and MD simulation studies. Along with the RBD Gamma, Delta, Beta, and Alpha variants, the United States (US/lineage B.1.429/Epsilon) variant and the WT were examined in this study.

### 2.1. Analysis of the Modeled RBD Structures

The 3D structures of all studied RBDs were modeled and minimized using the SWISS-MODEL web server. The results were validated using the PROCHECK service, and are presented in Ramachandran plots, as shown in Figure 2 and Table 1. The WT RBD (PDB ID: 6M0J) crystal structure served as a control. The Ramachandran plot was divided into four types of areas by ProCheck: most favored, additional allowed, generously allowed, and disallowed [42]. If the percentage of non-glycine residues in the disallowed region was <15%, the protein structure was considered to be of high quality: the lower the percentage, the higher the quality of the protein structure [43,44]. Based on the results of the analysis, five models of the structure of RBD variants could be accepted based on the non-glycine-residue data in the disallowed region. The percentage of amino-acid residues in the most favored region and the disallowed region indicated the quality of the structure based on its geometry: the greater the percentage of amino-acid residues in the most favored region and the lower the percentage of residues in the disallowed region, the better the quality of the structure.

### 2.2. Molecular Docking Analysis

**Figure 3:**
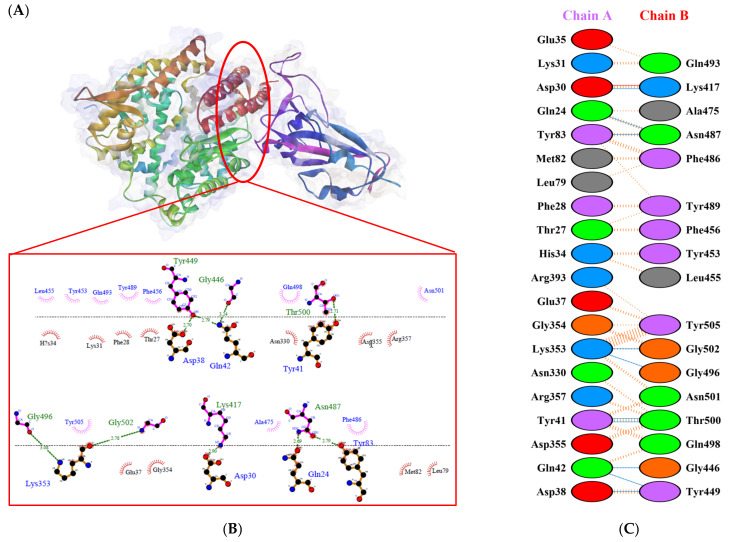
Protein–protein docking representation of hACE2 and RBD WT complexes. (**A**) The complexes binding interface; (**B**) The amino acids binding interaction; and (**C**) The interaction representation, which includes hydrogen (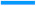), salt bridges (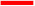), and non-bonded (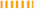) interactions. Chain A represents hACE2 and chain B represents RBD WT. Residue colors: positive (blue): H,K,R; negative (red): D,E; neutral (green): S,T,N,Q; aliphatic (grey): A,V,L,I,M; aromatic (purple): F,Y,W; Pro & Gly (orange): P,G.

**Figure 4:**
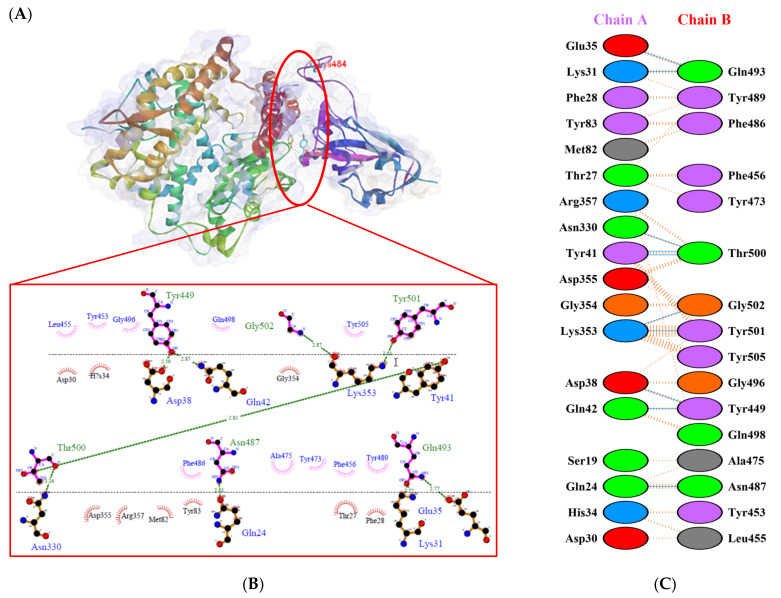
Protein–protein docking representation of hACE2 and RBD Gamma complexes. (**A**) The complex’s binding interface; (**B**) The amino acid’s binding interaction; and (**C**) The interaction representation, which includes hydrogen (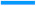), salt bridges (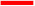), and non-bonded (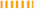) interactions. Chain A represents hACE2 and chain B represents RBD Gamma. Residue colors: positive (blue): H,K,R; negative (red): D,E; neutral (green): S,T,N,Q; aliphatic (grey): A,V,L,I,M; aromatic (purple): F,Y,W; Pro & Gly (orange): P,G.

**Figure 5:**
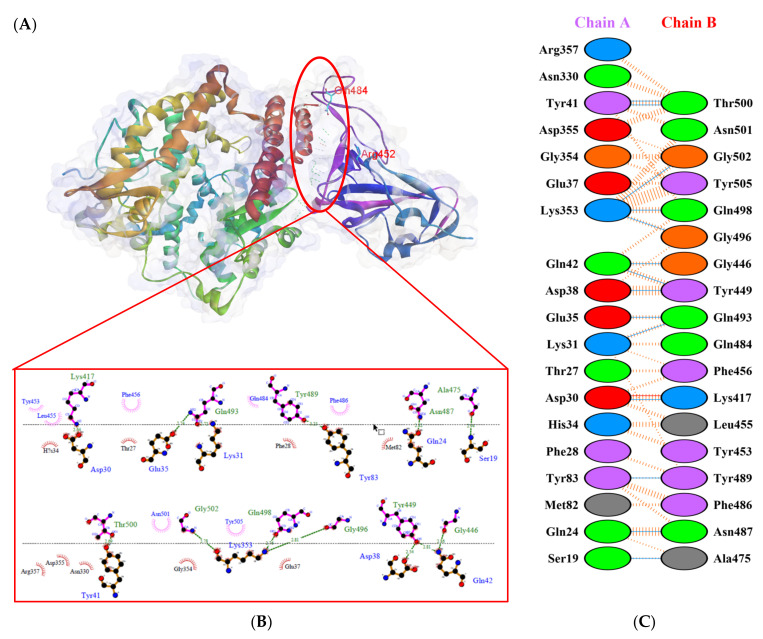
Protein–protein docking representation of hACE2 and RBD Delta complexes. (**A**) The complex’s binding interface; (**B**) The amino acid’s binding interaction; and (**C**) The interaction representation, which includes hydrogen (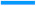), salt bridges (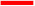), and non-bonded (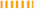) interactions. Chain A represents hACE2 and chain B represents RBD Delta. Residue colors: positive (blue): H,K,R; negative (red): D,E; neutral (green): S,T,N,Q; aliphatic (grey): A,V,L,I,M; aromatic (purple): F,Y,W; Pro & Gly (orange): P,G.

**Figure 6:**
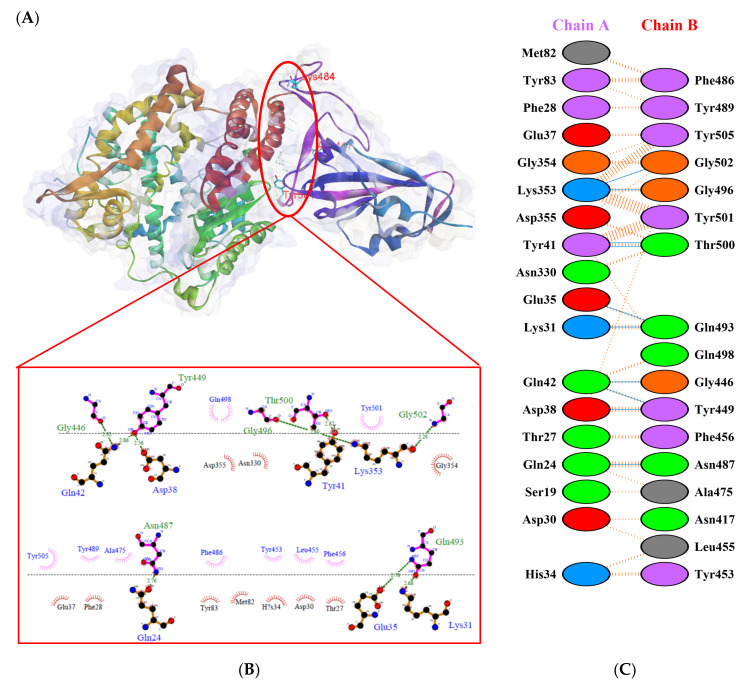
Protein–protein docking representation of hACE2 and RBD Beta complexes. (**A**) The complex’s binding interface; (**B**) The amino acid’s binding interaction; and (**C**) The interaction representation, which includes hydrogen (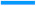), salt bridges (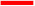), and non-bonded (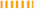) interactions. Chain A represents hACE2 and chain B represents RBD Beta. Residue colors: positive (blue): H,K,R; negative (red): D,E; neutral (green): S,T,N,Q; aliphatic (grey): A,V,L,I,M; aromatic (purple): F,Y,W; Pro & Gly (orange): P,G.

**Figure 7:**
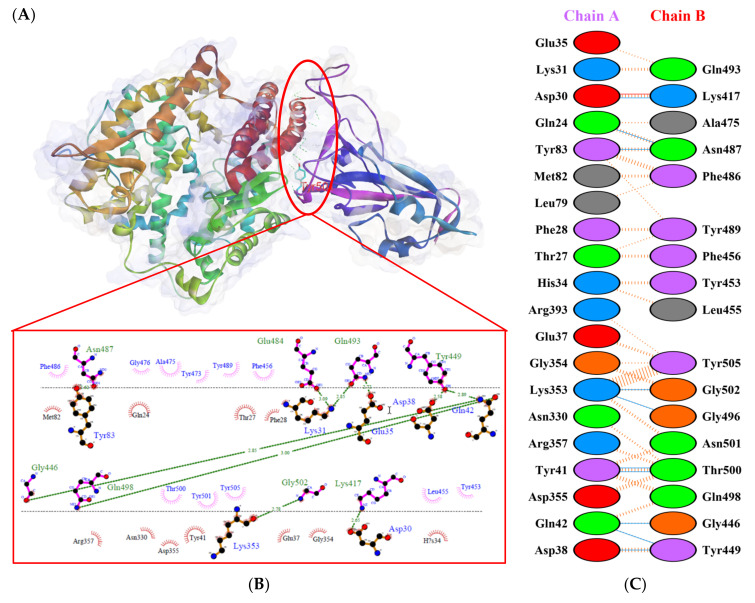
Protein–protein docking representation of hACE2 and RBD Alpha complexes. (**A**) The complex’s binding interface; (**B**) The amino acid’s binding interaction; and (**C**) The interaction representation, which includes hydrogen (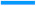), salt bridges (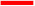), and non-bonded (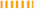) interactions. Chain A represents hACE2 and chain B represents RBD Alpha. Residue colors: positive (blue): H,K,R; negative (red): D,E; neutral (green): S,T,N,Q; aliphatic (grey): A,V,L,I,M; aromatic (purple): F,Y,W; Pro & Gly (orange): P,G.

**Figure 8:**
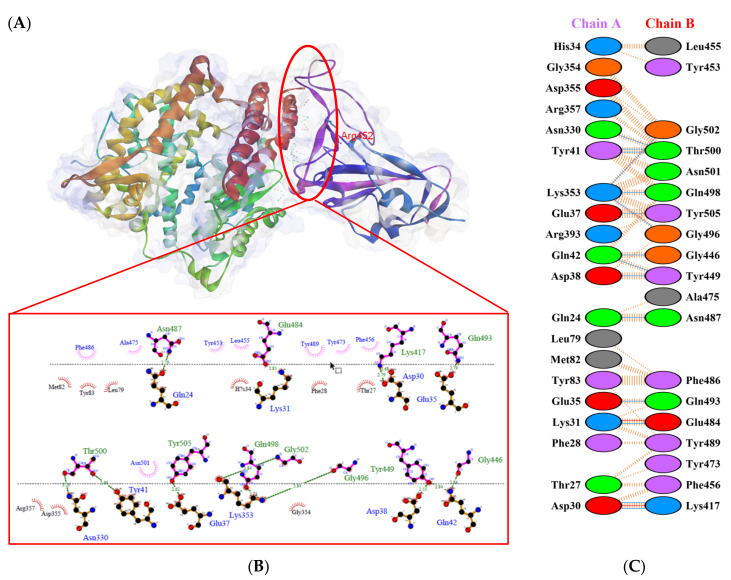
Protein–protein docking representation of hACE2 and RBD Epsilon complexes. (**A**) The complex’s binding interface; (**B**) The amino acid’s binding interaction; and (**C**) The interaction representation, which includes hydrogen (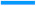), salt bridges (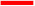), and non-bonded (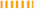) interactions. Chain A represents hACE2 and chain B represents RBD Epsilon. Residue colors: positive (blue): H,K,R; negative (red): D,E; neutral (green): S,T,N,Q; aliphatic (grey): A,V,L,I,M; aromatic (purple): F,Y,W; Pro & Gly (orange): P,G.

Protein-protein docking uses the biomolecular interaction between two molecules to determine the strength of a complex. Protein-protein docking simulation of hACE2 and RBD variants was performed on the HDOCK webserver. The results are visualized as illustrated in Figure 3, Figure 4, Figure 5, Figure 6, Figure 7 and Figure 8. The Biovia Discovery Studio was used to generate 3D visualizations, while LigPlot+ and PDBsum were used to generate 2D visualizations.

A previous study by Khan et al. [31] showed that hACE2–RBD forms hydrogen bonds (H-bonds) with Glu30–Lys417, Glu35–Gln493, Glu38–Tyr449, Glu38–Gly496, Tyr41–Thr500, Tyr41–Thr500, Gln42–Gln498, Asn330–Thr500, Lys353–Gly502, Lys353–Gly496, and Lys353–Gln498, as well as a salt bridge between Glu30 and Lys417. Several other researchers also confirmed that the following residues were important in RBD attachment to hACE: Gly446, Tyr449, Leu455, Phe486, Gln493, Gly496, Gln498, Thr500, Asn501, and Gly502 [45,46,47]. Moreover, Phe486, Gln493, and Asn501 in RBD were the most important residues identified by the hACE2 receptor on infected human cells, which simplified the RBD–hACE2 interaction [31,48].

As shown in Table 2, the present analysis of the interaction revealed the formation of H-bonds between hACE2 and residues in the active site of RBD Gamma: specifically, Glu35–Gln493 [O...H-N], Lys31–Gln493 [N-H...O], Asn330–Thr500 [N-H...O], Tyr41–Thr500 [O-H...O], Lys353–Gly502 [O...H-N], Asp38–Tyr449 [O...H-O], Gln42–Tyr449 [N...H-O], with distances of 2.77 Å, 2.72 Å, 3.24 Å, 2.81 Å, 2.87 Å, 2.59 Å, and 2.87 Å, respectively. Furthermore, the interaction of hACE2 on RBD Delta formed H-bonds as follows: Tyr41–Thr500 [O-H...O], Lys353–Gly502 [O...H-N], Lys353–Gln498 [N-H...O], Lys353–Gly496 [N-H...O], Gln42–Gly446 [N-H...O], Gln42–Tyr449 [N...H-O], Asp38–Tyr449 [O...H-O], Glu35–Gln493 [O...H-N], Lys31–Gln493 [N-H...O], and Asp30–Lys417 [O...H-N], with respective distances of 2.69 Å, 2.78 Å, 3.76 Å, 2.81 Å, 2.86 Å, 2.81 Å, 2.74 Å, 2.72 Å, and 2.64 Å. In the interaction between hACE2 and RBD Beta, H-bonds were detected in Lys353–Gly502 [O...H-N], Lys353–Gly496 [N-H...O], Tyr41–Thr500 [O-H...O], Glu35–Gln493 [O...H-N], Lys31–Gln493 [N-H...O], Gln42–Gly446 [N-H...O], Gln42–Tyr449 [N...H-O], and Asp38–Tyr449 [O...H-O] with distances of 3.29 Å, 2.80 Å, 2.82 Å, 2.78 Å, 2.68 Å, 2.92 Å, 2.86 Å, and 2.56 Å, respectively.

The following H-bonds were formed in the interaction of hACE2 and RBD Alpha: Asp30–Lys417 [O...H-N], Lys353–Gly502 [O...H-N], Gln42–Gly446 [N-H...O], Gln42–Tyr449 [N...H-O], and Asp38–Tyr449 [O...H-O], with respective distances of 2.65 Å, 2.78 Å, 2.85 Å, 2.89 Å, and 2.73 Å. The hACE2–RBD Epsilon interaction formed H-bonds as follows: Asn330–Thr500 [N-H...O], Tyr41–Thr500 [O-H...O], Lys353–Gly502 [O...H-N], Lys353–Gln498 [N-H...O], Lys353–Gly496 [N-H...O], Gln42–Gly446 [N-H...O], Gln42–Tyr449 [N...H-O], Asp38–Tyr449 [O...H-O], Gln24–Asn487 [O...H-N], Glu35–Gln493 [O...H-N], and Asp30–Lys417 [O...H-N], with distances of 3.29 Å, 2.68 Å, 2.84 Å, 2.72 Å, 2.84 Å, 2.84 Å, 2.84 Å, 2.55 Å, 2.79 Å, 2.79 Å, and 2.68 Å. Finally, the interaction of hACE2 and RBD WT resulted in the formation of H-bonds at residues Asp30–Lys417 [O...H-N], Gln24–Asn487 [O...H-N], Tyr83–Asn487 [O-H...O], Lys353–Gly502 [O...H-N], Lys353–Gly496 [N-H...O], Tyr41–Thr500 [O-H...O], Gln42–Gly446 [N-H...O], Gln42–Tyr449 [N...H-O], and Asp38–Tyr449 [O...H-O], with distances of 2.90 Å, 2.69 Å, 2.79 Å, 2.78 Å, 3.08 Å, 2.71 Å, 3.24 Å, 2.79 Å, and 2.70 Å, respectively. The bonds that formed between hACE2 and all of the RBDs were Asp38–Tyr449, Gln42–Tyr449, and Lys353–Gly502.

Considering the three residues required for the formation of the hACE2–RBD interaction, the Gamma and Beta variants had hydrogen bonds on the Gln493 residue, while Phe486 had hydrophobic contact. The H-bond was located on the Gln493 residue in the Delta and Epsilon variants, while the hydrophobic contacts were located on Phe486 and Asn501. In the Alpha and WT variants, the three residues showed hydrophobic contact alone. Of the six variants analyzed, four showed H-bonding at the Gln493 residue while two variants showed hydrophobic contact. Six variants made only hydrophobic contact with the Phe486 residue, whereas four variants made hydrophobic contact with Asn501. Salt bridges were formed during the interaction of hACE2 with RBD WT Asp30–Lys417 (2.90), Delta Asp30–Lys417 (2.64), Epsilon Asp30–Lys417 (2.68) and Lys31–Glu484 (2.85), Alpha Asp30–Lys417 (2.65), and Lys31–Glu484 (2.85). 

### 2.3. Molecular Dynamics Simulation of Protein–Protein Complexes 

**Figure 9:**
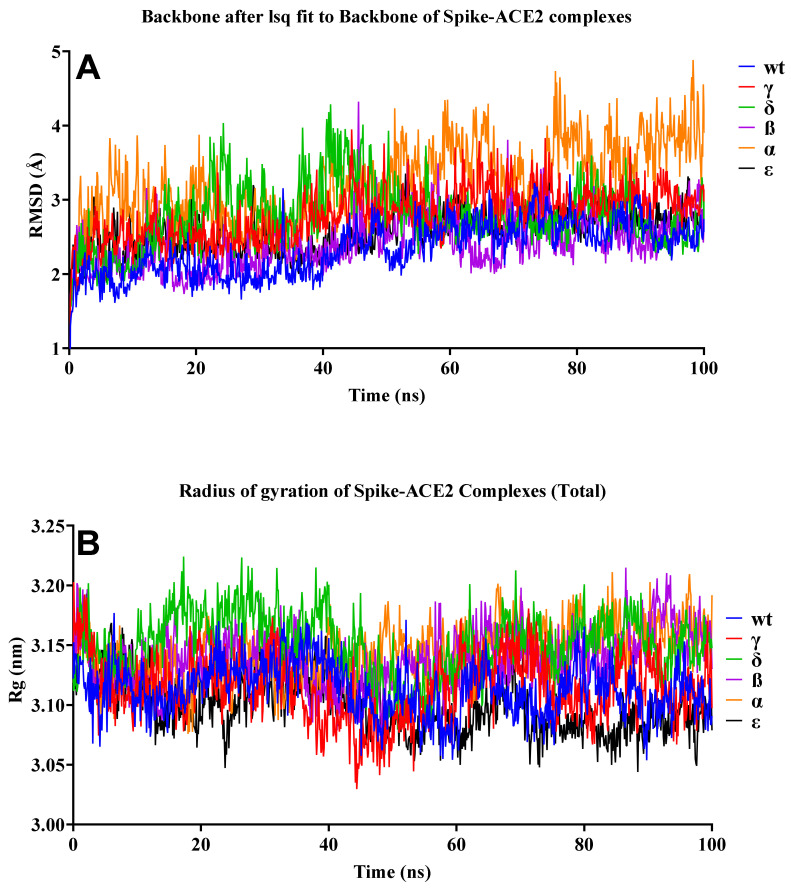
RMSD and Rg of the S–hACE2 complexes. (**A**) RMSD change in WT and mutant S–hACE2 complex backbones during 100-ns MD simulation. (**B**) Total Rg change over time.

The stability and pattern of binding of protein-protein interactions (PPIs) change according to physiological circumstances and time [49]. To provide insight into the dynamic status and estimation of different bond forms between two proteins, long-term molecular dynamics (MD) simulation of the selected protein-protein binding complexes (hACE2 and RBD) were performed. While examining the structural stability of the protein complexes during the 100-ns MD simulation, it was discovered that the stable structure was preserved, as illustrated in Figure 9. The Alpha variant was the least stable complex, with a fluctuation of up to 4 Å. By contrast, the results for the Rg in all structures indicated that structural stability did not deteriorate during the MD simulation. MD simulations revealed that SARS-CoV-2 VOCs interacted more strongly with the host receptor hACE2. This finding was consistent with prior research indicating that the N501Y mutation, which affected the primary contact residue in RBD, increased the viral attachment proclivity for hACE2 [50,51]. The combination of mutations S477N/E484K, E484K/N501Y, and K417T/E484K/N501Y increased viral affinity for hACE2 and also antibody resistance [52].

**Figure 10:**
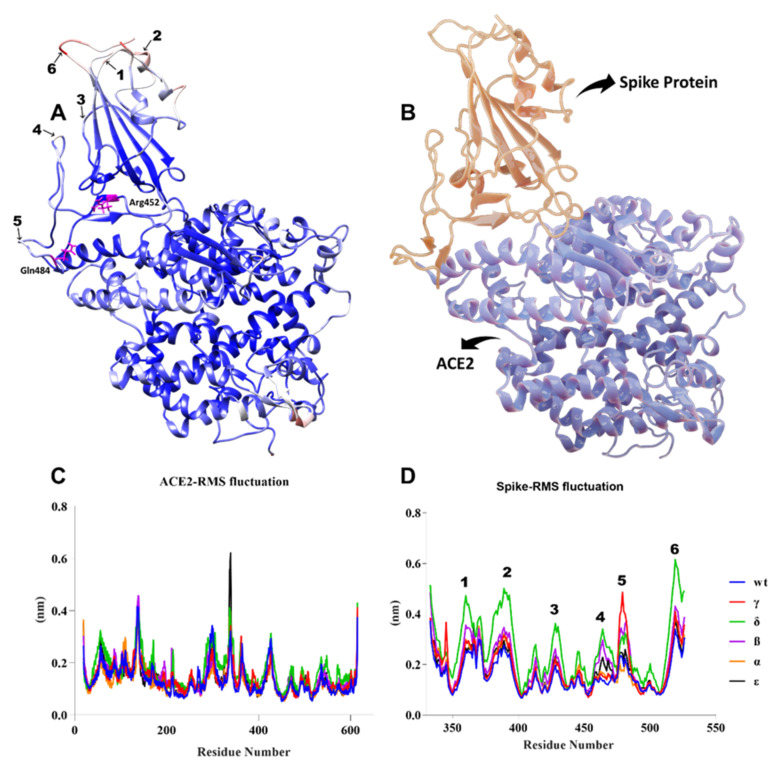
(**A**) B-factor representation of delta variant S-hACE2 complex residues during 100-ns MD simulation (red represents high fluctuations and blue represents low fluctuations). Mutant residues are indicated in pink. (**B**) S-protein interaction with hACE2 receptor (red represents S protein and blue represents ACE2 receptor). (**C**,**D**) RMSF of ACE2 and S residues throughout the 100-ns MD simulation.

As illustrated in Figure 10A, the most flexible regions during the 100-ns simulation were the terminal parts of S proteins. While no significant change was observed in the ACE2 protein compared to the WT during the simulation, we observed significant conformational changes in the S protein, especially in the Delta variant. As seen in Figure 10C,D, we observed residual fluctuations in areas 1, 2, 3, 4, and 6. This might have signified that the immune response was delayed, as the antibody recognition sites may also have changed. Recent studies have shown that vaccine efficacy was lower in the Delta variant, which was consistent with our findings [53]. While no significant fluctuation was observed in other variants, it was clearly seen in residues in the range of 477 to 480 in the Gamma variant. 

As the hydrogen-bond stability of the complexes was examined during the 100-ns MD simulation, the Tyr83-side/Asn487-side, Gln493-side/Glu35-side, Thr500-side/Asp355-side, and Gly502-main/Lys353-main hydrogen bonds, which played a role in the binding of the S to hACE2 proteins, were found to be critical in all variants (Table 3). This was consistent with the discovery of Khan et al. [31] that hACE2–RBD formed hydrogen bonds between Glu35–Gln493 and Lys353–Gly502. However, it was observed that hydrogen-bond formation between Tyr505-side/Glu37-side occurred at a high rate in WT and the Epsilon variant. The Ser19-side and Ala475-main H-bonds, which played a role in the formation of the complex, were effective in the WT, Gamma, and Delta variants, but not in the others. Ashwaq et al. [54] reported similar findings.

Notably, the hydrogen bond between Tyr449-side and Asp38-side was ineffective in the WT but highly effective in the other variants. While a hydrogen bond between Tyr501-side and Asp38-side was observed in the Gamma variant, this interaction did not occur in the others. A hydrogen bond was established between Gln498-side and Asp355-side in the Delta variant; However, this formation was not observed in the other variants.

### 2.4. Binding Free Energy of Protein–Protein Complex Simulation Trajectory

**Figure 11:**
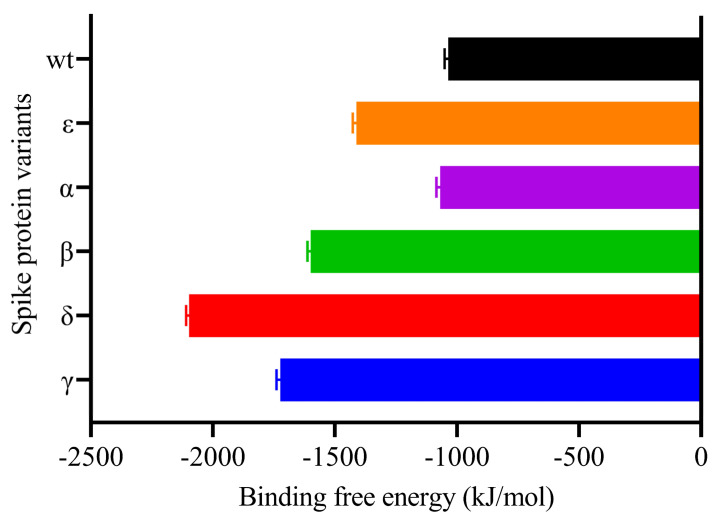
MM-PBSA binding free energy estimation of Spike-ACE2 complexes.

As the binding free energies of the S protein variants with hACE2 were examined, we observed the strongest result in the Delta variant (Table 4, and Figure 11). Recent investigations have demonstrated that the Delta variant is more effective than others at binding to hACE2 [55]. Additionally, this variation appears to spread at a considerably faster rate than the other three VOCs [25]. Residue 478, which is located in the flexible loop, is most likely to come into contact with ACE2. This appears to strengthen the interaction between the Delta variant and ACE2, which accounts for the dramatic increase in the proportion of infectivity of this variant [56]. The interaction between RBD WT and hACE2 exhibited the lowest binding affinity. This implied that the existence of mutations in RBD resulted in a greater binding affinity for hACE2. Laffeber et al. [57] demonstrated experimentally that RBD carrying the N501Y mutation had a sevenfold greater affinity for the hACE2 receptor than WT RBD. However, it was reported that mutations at the position K417N/T decreased the binding affinity [58]. Furthermore, a K-to-N mutation significantly reduced the binding affinity between N417/Y501-RBD and ACE2 when compared to the Y501-RBD to ACE interaction [59].

**Figure 12:**
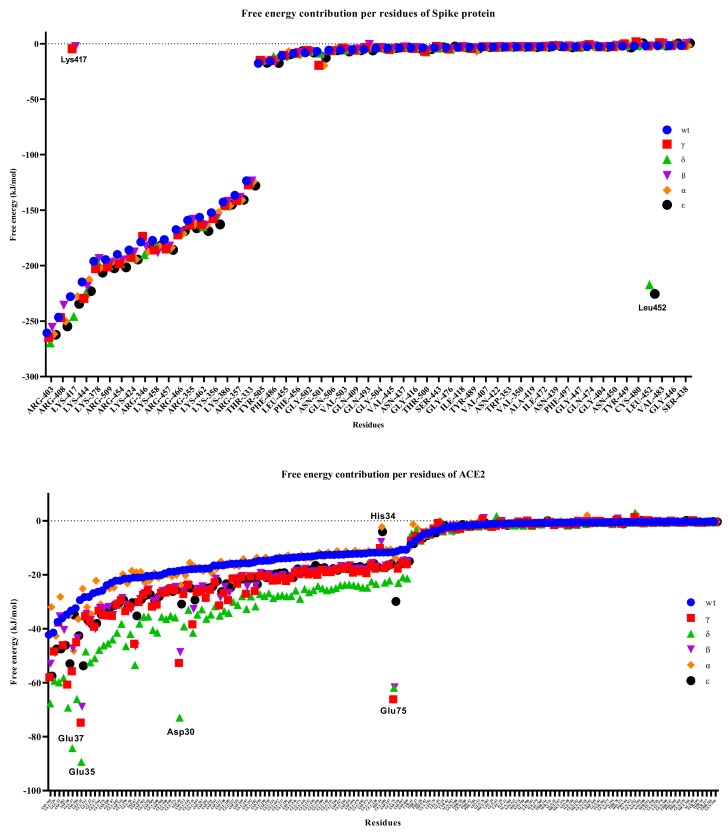
Free energy contributions per residue of S-hACE2 complexes throughout 100 ns MD simulation.

As the residual contribution to the binding free energies was examined, no significant changes were observed in the S protein variants, but significant differences were observed in the hACE2 protein (Figure 12). We observed that many residues of the S protein had lower binding free energies than the hACE2 residues, with the most significant change being observed in the interaction of the Delta variant. In particular, we calculated that Asp30, Glu35, and Glu37 in hACE2 were significantly separated from the others as the residues with the lowest binding free energy in the Delta variant. We observed that the His34 residue had high binding free energy in the Alpha, Epsilon, and Beta variants, while the value for Glu75 was significantly lower in the Gamma, Delta, and Beta variants. Chakraborty et al. [60] discovered that His34 of ACE2 had the second highest energetic contribution (4.68 kcal/mol) when compared to Arg403 of RBD via a direct hydrogen bond.

While it was observed that Lys417, one of the S protein residues, was ineffective in the Gamma and Beta variants, we found that Leu452 in the Delta and Epsilon variants had a relatively low binding free energy of <200 kJ/mol and was largely dissociated from the other variants. In the WT RBD, Lys417 formed a salt bridge with Glu30 of RBD [31]. Salt bridges, like disulfide bonds, can act as keystone interactions [61].

## 3. Materials and Methods

### 3.1. Preparation of the Macromolecules

The three-dimensional (3D) crystal structure of WT SARS-CoV-2 S RBD bound with hACE2 was retrieved from the Protein Data Bank (https://www.rcsb.org/ (accessed on 26 May 2021)) with PDB ID 6M0J. The 3D structures of the RBD variants were modeled and minimized using the SWISS-MODEL web server (https://swissmodel.expasy.org/ (accessed on 28 May 2021)) [62,63]. The structural accuracy of the RBD protein model was analyzed using the PROCHECK server (https://saves.mbi.ucla.edu/ (accessed on 28 May 2021)) [64]. The analytical results are presented in a Ramachandran plot.

### 3.2. Molecular Docking Assay

The HDOCK server (http://hdock.phys.hust.edu.cn/ (accessed on 30 May 2021)) was used to perform the protein–protein docking analysis [65,66]. This server uses a hybrid algorithm of template-based and template-free docking to predict interactions between hACE2 and RBD. The 3D interactions were created using the BIOVIA Discovery Studio Visualizer version 21.1.0, while the 2D interactions were created using LigPlot+ version 4.5.3 [67] and PDBsum (http://www.ebi.ac.uk/thornton-srv/databases/pdbsum (accessed on 3 June 2021)) [68].

### 3.3. Molecular Dynamics (MD) Simulations

All simulations were run via GROMACS 2021.2 software using the Leap-Frog integration at 2-fs intervals [69]. The MD simulation system was built under periodic boundary conditions (PBCs) with the ‘rhombic dodecahedron’ scheme. The distance from the protein complex to the corner of the cube was set to 1.2 nm. Amber99SB-ildn was chosen as the force field and ‘TIP3P’ was preferred as the water model [70]. The system was neutralized by adding 0.15 mM NaCl. Energy minimization was done by the Steepest Descent algorithm in 50,000 steps (minimization stopped when the maximum force was <10.0 kJ/mol). To bring the MD system to the equilibrium phase, 100-ps NVT (constant number of particles, volume, and temperature) and 1-ns NPT (constant number of particles, pressure, and temperature) simulations were performed. In the NVT and NPT stages, all bonds and heavy atoms were restricted by the LINCS (LINear Constraint Solver) algorithm. In the NVT phase, the V-rescale coupling algorithm was the preferred temperature coupling algorithm. In the NPT phase, V-rescale was preferred as the temperature coupling algorithm, and the Parrinello-Rahman with isothermal compressibility was preferred as the pressure coupling algorithm. The temperature and pressure were set to 310 K and 1 atm, respectively. In the MD production phase, unlike the NPT phase, the atomic restrictions were removed and a 100-ns MD simulation was carried out. The Verlet algorithm was used as the cutoff scheme. The Particle-Mesh Ewald (PME) method was preferred for long-range interactions. The cutoff values of the electrostatic and Van der Waals interactions were both set to 1.2 nm.

### 3.4. Analysis of MD Simulations

The root-mean-square deviation (RMSD) and hydrogen bond analyses were performed with the VMD program (ref). The root-mean-square fluctuation (RMSF) and radius of gyration (Rg) analyses were performed with the Gromacs RMSF version 2019.2 and gyrate tools, respectively. All analyses were plotted using GraphPad Prism version 9.1.2 for Windows (GraphPad Software, San Diego, CA, USA; www.graphpad.com (accessed on 10 June 2021)).

### 3.5. Free-Energy Calculations

The binding free energies were calculated using the molecular mechanics Poisson–Boltzmann surface area (MMPBSA) approach, which is an end-point method [71]. The binding free energies, including the entropy contribution, were obtained by taking 100 snapshots at 100-ps intervals in the last 10 ns. The polar component of desolvation was calculated using PB models. In the PB calculations, the partial charges of the proteins were taken from the forcefield parameters. The solvent-accessible surface area (SASA) was preferred as a non-polar contribution. The vacuum electrostatic dielectric constant and the solvent dielectric constant were set to 2 and 80, respectively. The g_mmpbsa tool was used for the MMPBSA calculation [72]. The binding free-energy calculations were made according to the following equation:

ΔG_Binding_ = G_complex_ − (G_protein_ + G_ligand_) (1)

ΔG_Solv_ = ΔG_polar_ + ΔG_nonpolar_ (2)

## 4. Conclusions

The five variants of the RBD of SARS-CoV-2 S-proteins had distinct mutations at their binding sites with hACE2. The present study investigated the PPIs of mutant RBD (K417N, E484K, and N501Y in B.1.351 (Beta); L452R and E484Q in B.1.617 (Delta); K417T, E484K, and N501Y in P.1 (Gamma); L452R in B.1.429; and N501Y in B.1.1.7 (Alpha)) with hACE2. The increased binding affinity of B.1.617 (IN), P.1 (BR), and B.1.351 (SA), (CA) strain with hACE2 when compared to WT indicated the possibility of high transmissibility and rapid spread. Calculation of the binding free energy of the protein-protein complex revealed that the Delta variant had the lowest value, indicating that it bound to hACE2 more strongly than the others. Furthermore, it was predicted that mutations in the B.1.617 (IN)/Delta variant, which caused more residual fluctuations than other variants, may play a role in antibody escape. The MD simulation analysis demonstrated a stable interaction between RBD variants and hACE2. This is expected to have an effect on drug discovery efforts by elucidating residues that could be targeted for disrupting this interface. Overall, this study laid the groundwork for the development of antibodies, vaccines, and drugs against new SARS-CoV-2 variants.

## Figures and Tables

**Table 1:** Ramachandran statistics for the predicted model.

Variants	Most Favored Region (%)	Additional Allowed Region (%)	Generously Allowed Region (%)	Disallowed Region (%)
Wild-type *	89.3	10.7	0.0	0.0
Gamma	90.5	9.5	0.0	0.0
Delta	90.5	9.5	0.0	0.0
Beta	92.3	7.7	0.0	0.0
Alpha	91.7	8.3	0.0	0.0
Epsilon	90.5	9.5	0.0	0.0

* SARS-CoV-2 wild-type RBD (PDB ID:6MJ0).

**Table 2:** The H-bonds between hACE2 and the active site of RBD.

hACE2 Residues	RBD Residues					
	WT	Gamma	Delta	Beta	Alpha	Epsilon
Ser19	-	-	Ala475	-	-	-
Gln24	Asn487	Asn487	Asn487	Asn487	-	Asn487
Asp30	Lys417	-	Lys417	-	Lys417	Lys417
Lys31	-	-	-	-	Glu484	Glu484
	-	Gln493	Gln493	Gln493	Gln493	-
Glu35	-	Gln493	Gln493	Gln493	Gln493	Gln493
Glu37	-	-	-	-	-	Tyr505
Asp38	Tyr449	Tyr449	Tyr449	Tyr449	Tyr449	Tyr449
Tyr41	Thr500	Thr500	Thr500	Thr500	-	Thr500
Gln42	Gly446	-	Gly446	Gly446	Gly446	Gly446
	Tyr449	Tyr449	Tyr449	Tyr449	Tyr449	Tyr449
	-	-	-	-	Gln498	-
Tyr83	Asn487	-	-	-	Asn487	-
	-	-	Tyr489	-	-	-
Asn330	-	Thr500		-	-	Thr500
Lys353	Gly496	-	Gly496	Gly496	-	Gly496
	-	-	Gln498	-	-	Gln498
	-	Tyr501	-	-	-	-
	Gly502	Gly502	Gly502	Gly502	Gly502	Gly502

**Table 3:** Hydrogen-bond stabilities during the 100-ns MD simulation of S–hACE2 complexes.

Hydrogen Bonds		WT	Gamma	Delta	Beta	Alpha	Epsilon
Donor	Acceptor	Occupancy					
Tyr83-side	Asn87-side	49.75%	47.95%	46.95%	48.85%	-	47.65%
Gln493-side	Glu35-side	47.05%	47.35%	47.65%	43.16%	-	42.96%
Tyr505-side	Glu37-side	45.85%	9.39%	2.20%	7.49%	-	45.55%
Thr500-side	Asp355-side	33.47%	53.35%	37.16%	46.05%	-	26.77%
Gly502-main	Lys353-main	30.77%	44.66%	28.47%	48.55%	-	40.56%
Lys417-side	Asp30-side	27.97%	-	10.29%	-	30.67%	27.27%
Ser19-side	Ala475-main	26.67%	33.87%	36.26%	1.90%	2.50%	4.20%
Lys31-side	Gln493-side	20.88%	7.99%	12.69%	8.39%	-	9.89%
Thr500-side	Tyr41-side	18.98%	4.60%	13.09%	5.79%	-	26.17%
Tyr489-side	Tyr83-side	18.88%	-	-	-	-	-
Lys353-side	Tyr495-main	13.19%	-	4.90%	0.20%	-	16.48%
Tyr449-side	Asp38-side	3.00%	35.76%	48.95%	43.16%	27.27%	60.04%
Tyr501-side	Asp38-side	-	14.89%	-	-	-	-
Ser477-side	Glu23-side	-	10.99%	-	1.70%	-	-
Tyr505-side	Ala386-main	-	-	19.68%	1.70%	-	-
Gln498-side	Asp355-side	-	-	15.88%	-	-	-
Tyr453-side	His34-side	-	0.30%	0.10%	32.97%	3.50%	0.20%

Darker green indicates that the H-bonds remain stable for a higher percentage of the time, while darker yellow indicates that H-bonds remain stable for a lower percentage of the time.

**Table 4:** MMPBSA free-energy analysis of S-protein variants and hACE2 binding.

	Van der Waal Energy kJ/mol	Electrostatic Energy kJ/mol	Polar Solvation Energy kJ/mol	SASA Energy kJ/mol	Binding Energy kJ/mol
WT	−383,845 (2.17)	−1,427,047 (7.577)	820,013 (18.49)	−45,734 (0.374)	−1,036,072 (14.875)
Gamma	−395,679 (2.335)	−1,757,205 (5.821)	473,056 (14.625)	−4463 (0.335)	−1,723.82 (15.445)
Delta	−371,792 (2.857)	−2,384,008 (6.377)	705,859 (12.583)	−46,789 (0.346)	−2,097,241 (11.978)
Beta	−379,902 (2.127)	−1,718,065 (7.052)	542,671 (12.887)	−44,012 (0.403)	−1,599,527 (12.756)
Alpha	−366.51 (2.228)	−1,358,149 (9.501)	701,388 (16.644)	−4414 (0.371)	−1,069.033 (15.474)
Epsilon	−364,326 (2.824)	−1972,844 (7.259)	970,525 (13.802)	−4525 (0.394)	−1412,592 (14.606)

## Data Availability

The authors confirm that the data supporting the findings of this study are available within the article. Raw data were generated on webservers provided in the Section 3. Derived data supporting the findings of this study are available from the corresponding author on request.

## References

[1] Lai C.C., Shih T.P., Ko W.C., Tang H.J., Hsueh P.R. (2020). Severe acute respiratory syndrome coronavirus 2 (SARS-CoV-2) and coronavirus disease-2019 (COVID-19): The epidemic and the challenges. Int. J. Antimicrob. Agents.

[2] Tallei T.E., Tumilaar S.G., Niode T.J., Fatimawali F., Kepel B.J., Idroes R., Effendi Y., Sakib S.A., Emran T.B. (2020). Potential of plant bioactive compounds as SARS-CoV-2 main protease (Mpro) and spike (S) glycoprotein inhibitors: A molecular docking study. Scientifica.

[3] Sharun K., Tiwari R., Dhama K., Emran T.B., Rabban A.A., Al Mutair A. (2021). Emerging SARS-CoV-2 variants: Impact on vaccine efficacy and neutralizing antibodies. Hum. Vaccines Immunother..

[4] Romano M., Ruggiero A., Squeglia F., Maga G., Berisio R. (2020). A structural view of SARS-CoV-2 RNA replication machinery: RNA synthesis, proofreading and final capping. Cells.

[5] Khairan K., Idroes R., Tallei T.E., Nasim M.J., Jacob C. (2021). Bioactive compounds from medicinal plants and their possible effect as therapeutics agents against COVID-19: A review. Curr. Nutr. Food Sci..

[6] Rakib A., Arkajyoti P., Uddin N.C., Sami S.A., Baral S.K., Majumder M., Tareq A.M., Amin M.N., Shahriar A., Uddin Z. (2020). Biochemical and computational approach of selected phytocompounds from *Tinospora crispa* in the management of COVID-19. Molecules.

[7] Yadav R., Chaudhary J.K., Jain N., Chaudhary P.K., Khanra S., Dhamija P., Sharma A., Kumar A., Hadu S. (2021). Role of structural and non-structural proteins and therapeutic targets of SARS-CoV-2 for COVID-19. Cells.

[8] Chen N., Zhou M., Dong X., Qu J., Gong F., Han Y., Qiu Y., Wang J., Liu Y., Wei Y. (2020). Epidemiological and clinical characteristics of 99 cases of 2019 novel coronavirus pneumonia in Wuhan, China: A descriptive study. Lancet.

[9] Ceraolo C., Giorgi F.M. (2020). Genomic variance of the 2019-nCoV coronavirus. J. Med. Virol..

[10] Naqvi A.A.T., Fatima K., Mohammad T., Fatima U., Singh I.K., Singh A., Atif S.M., Hariprasad G., Hasan G.M., Hassan I. (2020). Insights into SARS-CoV-2 genome, structure, evolution, pathogenesis and therapies: Structural genomics approach. Biochim. Biophys. Acta Mol. Basis Dis..

[11] Li W., Moore M.J., Vasilieva N., Sui J., Wong S.K., Berne M.A., Somasundaran M., Sullivan J.L., Luzuriaga K., Greenough T.C. (2003). Angiotensin-converting enzyme 2 is a functional receptor for the SARS coronavirus. Nature.

[12] Ou X., Liu Y., Lei X., Li P., Mi D., Ren L., Guo L., Guo R., Chen T., Hu J. (2020). Characterization of spike glycoprotein of SARS-CoV-2 on virus entry and its immune cross-reactivity with SARS-CoV. Nat. Commun..

[13] Papa G., Mallery D.L., Albecka A., Welch L.G., Cattin-Ortola J., Luptak J., Paul D., McMahon H.T., Goodfellow I.G., Carter A. (2021). Furin cleavage of SARS-CoV-2 Spike promotes but is not essential for infection and cell-cell fusion. PLoS Pathog..

[14] Henderson R., Edwards R.J., Mansouri K., Janowska K., Stalls V., Gobeil S., Kopp M., Hsu A., Borgnia M., Parks R. (2020). Controlling the SARS-CoV-2 Spike glycoprotein conformation. bioRxiv Prepr. Serv. Biol..

[15] Huang Y., Yang C., Xu X.F., Xu W., Liu S.W. (2020). Structural and functional properties of SARS-CoV-2 spike protein: Potential antivirus drug development for COVID-19. Acta Pharmacol. Sin..

[16] Faria N.R., Mellan T.A., Whittaker C., Claro I.M., da Candido D.S., Mishra S., Crispim M.A.E., Sales F.C., Hawryluk I., McCrone J.T. (2021). Genomics and epidemiology of a novel SARS-CoV-2 lineage in Manaus, Brazil. medRxiv Prepr. Serv. Health Sci..

[17] Madhi S.A., Baillie V., Cutland C., Voysey M., Phil D., Koen A.L., Fairlie L., Paeds F.C., Padayachee S.D., Dheda K. (2021). Efficacy of the ChAdOx1 nCoV-19 Covid-19 vaccine against the B.1.351 variant. N. Engl. J. Med..

[18] Noh J.Y., Jeong H.W., Shin E.-C. (2021). SARS-CoV-2 mutations, vaccines, and immunity: Implication of variants of concern. Signal Transduct. Target. Ther..

[19] Wang Z., Schmidt F., Nussenzweig M.C. (2021). mRNA vaccine-elicited antibodies to SARS-CoV-2 and circulating variants. Nature.

[20] Liu Z., VanBlargan L.A., Bloyet L.-M., Rothlauf P.W., Chen R.E., Stumpf S., Zhao H., Errico J.M., Theel E.S., Liebeskind M.J. (2021). Identification of SARS-CoV-2 spike mutations that attenuate monoclonal and serum antibody neutralization. Cell Host Microbe.

[21] Wang P., Wang M., Yu J., Cerutti G., Nair M.S., Huang Y., Kwong P.D., Shapiro L., Ho D.D. (2021). Increased resistance of SARS-CoV-2 variants B.1.351 and B.1.1.7 to Antibody neutralization. bioRxiv Prepr. Serv. Biol..

[22] Buchan B.W., Yao J.D. (2021). Severe acute respiratory syndrome Coronavirus 2: The emergence of important genetic variants and testing options for clinical laboratories. Clin. Microbiol. Newsl..

[23] Ramanathan M., Ferguson I.D., Miao W., Khavari P.A. (2021). SARS-CoV-2 B.1.1.7 and B.1.351 spike variants bind human ACE2 with increased affinity. Lancet Infect. Dis..

[24] Wang P., Casner R.G., Nair M.S., Wang M., Yu M., Cerutti G., Liu L., Kwong P.D., Huang Y., Shapiro L. (2021). Increased resistance of SARS-CoV-2 variant P.1 to antibody neutralization. Cell Host Microbe.

[25] Davies N.G. (2021). Estimated transmissibility and impact of SARS-CoV-2 lineage B.1.1.7 in England. Science.

[26] Tang J.W., Tambyah P.A., Hui D.S. (2021). Emergence of a new SARS-CoV-2 variant in the UK. J. Infect..

[27] Janik E., Niemcewicz M., Podogrocki M., Majsterek I., Bijak M. (2021). The emerging concern and interest SARS-CoV-2 variants. Pathogens.

[28] Tegally H., Wilkinson E., Giovanetti M., Iranzadeh A., Fonseca V., Giandhari J., Doolabh D., Pillay S., San E.J., Msomi N. (2020). Emergence and rapid spread of a new severe acute respiratory syndrome-related coronavirus 2 (SARS-CoV-2) lineage with multiple spike mutations in South Africa. medRxiv.

[29] Yadav P., Mohandas S., Sarkale P., Nyayanit D., Shete A., Sahay R., Potdar V., Baradkar S., Gupta N., Sapkal G. (2021). Isolation of SARS-CoV-2 B.1.1.28.2 P2 variant and pathogenicity comparison with D614G variant in hamster model. bioRxiv.

[30] Callaway E. (2021). Coronavirus variants get Greek names—But will scientists use them?. Nature.

[31] Khan A., Zia T., Suleman M., Khan T., Ali S.S., Abbasi A.A., Mohammad A., Wei D.-Q. (2021). Higher infectivity of the SARS-CoV-2 new variants is associated with K417N/T, E484K, and N501Y mutants: An insight from structural data. J. Cell. Physiol..

[32] Leung K., Shum M.H., Leung G.M., Lam T.T., Wu J.T. (2021). Early transmissibility assessment of the N501Y mutant strains of SARS-CoV-2 in the United Kingdom, October to November 2020. Eur. Surveill. Bull. Eur. Mal. Transm. Eur. Commun. Dis. Bull..

[33] Gobeil S.M.-C., Janowska K., McDowell S., Mansouri K., Parks R., Stalls V., Kopp M.F., Manne K., Li D., Wiehe K. (2021). Effect of natural mutations of SARS-CoV-2 on spike structure, conformation, and antigenicity. Science.

[34] Rees-Spear C., Muir L., Griffith S.A., Heaney J., Aldon Y., Snitselaar J.L., Thomas P., Graham C., Seow J., Lee N. (2021). The effect of spike mutations on SARS-CoV-2 neutralization. Cell Rep..

[35] Kirby T. (2021). New variant of SARS-CoV-2 in UK causes surge of COVID-19. Lancet. Respir. Med..

[36] Rakib A., Nain Z., Islam M.A., Sami S.A., Mahmud S., Islam A., Ahmed S., Siddiqui A.B.F., Babu S.M.O.F., Hossain P. (2021). A molecular modelling approach for identifying antiviral selenium-containing heterocyclic compounds that inhibit the main protease of SARS-CoV-2: An in silico investigation. Brief. Bioinform..

[37] Dutta M., Tareq A.M., Rakib A., Mahmud S., Sami S.A., Mallick J., Islam M.N., Majumder M., Uddin M.Z., Alsubaie A. (2021). Phytochemicals from *Leucas zeylanica* targeting main protease of SARS–CoV–2: Chemical profiles, molecular docking, and molecular dynamics simulations. Biology.

[38] Chowdhury K.H., Chowdhury R., Mahmud S., Tareq A.M., Hanif N.B., Banu N., Reza A.S.M., Emran T.B., Simal-Gandara J. (2021). Drug repurposing approach against novel coronavirus disease (COVID-19) through virtual screening targeting SARS-CoV-2 main protease. Biology.

[39] Li Q., Wu J., Nie J., Zhang L., Hao H., Liu S., Zhao C., Zhang Q., Liu H., Nie L. (2020). The impact of mutations in SARS-CoV-2 spike on viral infectivity and antigenicity. Cell.

[40] Weisblum Y., Schmidt F., Zhang F., DaSilva J., Poston D., Lorenzi J.C.C., Muecksch F., Rutkowska M., Hoffmann H.-H. (2020). Escape from neutralizing antibodies by SARS-CoV-2 spike protein variants. Elife.

[41] Singh A., Steinkellner G., Köchl K., Gruber K., Gruber C.C. (2021). Serine 477 plays a crucial role in the interaction of the SARS-CoV-2 spike protein with the human receptor ACE2. Sci. Rep..

[42] Kleywegt G.J., Jones T.A. (1996). Phi/Psichology: Ramachandran revisited. Structure.

[43] Lovell S.C., Ian W., Davis W., Arendall B., de Bakker P.I.W., Word M., Prisant M.G., Richardson J.S., Richardson D.C. (2003). Structure validation by Cα geometry: φ,ψ and Cβ deviation. Proteins Struct. Funct. Genet..

[44] Gowder S.M., Chatterjee J., Chaudhuri T., Paul K. (2014). Prediction and analysis of surface hydrophobic residues in tertiary structure of proteins. Sci. World J..

[45] Yan R., Zhang Y., Li Y., Xia L., Guo Y., Zhou Q. (2020). Structural basis for the recognition of SARS-CoV-2 by full-length human ACE2. Science.

[46] Lan J., Ge J., Yu J., Shan S., Zhou H., Fan S., Zhang Q., Shi X., Wang Q., Zhang L. (2021). Structure of the SARS-CoV-2 spike receptor-binding domain bound to the ACE2 receptor. Nature.

[47] Koley T., Ge J., Yu J., Shan S., Zhou H., Fan S., Zhang Q., Shi X., Wang Q., Zhang L. (2021). Structural analysis of COVID-19 spike protein in recognizing the ACE2 receptor of different mammalian species and its susceptibility to viral infection. 3 Biotech..

[48] Harapan H., Ryan M., Yohan B., Abidin R.S., Nainu F., Rakib A., Jahan I., Emran T.B., Ullah I., Panta K. (2021). COVID-19 and dengue: Double punches for dengue-endemic countries in Asia. Rev. Med. Virol..

[49] Peng X., Wang J., Peng W., Wu F.-X., Pan Y. (2017). Protein–protein interactions: Detection, reliability assessment and applications. Brief. Bioinform..

[50] Gu H., Chen Q., Zhou Y., Fan H., Deng Y.-Q., Wang Y., Teng Y., Zhao Z., Cui Y., Li Y. (2020). Adaptation of SARS-CoV-2 in BALB/c mice for testing vaccine efficacy. Science.

[51] Luan B., Wang H., Huynh T. (2021). Enhanced binding of the N501Y-mutated SARS-CoV-2 spike protein to the human ACE2 receptor: Insights from molecular dynamics simulations. FEBS Lett..

[52] Gan H.H., Twaddle A., Marchand B., Gunsalus K.C. (2021). Structural modeling of the SARS-CoV-2 spike/human ACE2 complex interface can identify high-affinity variants associated with increased transmissibility. J. Mol. Biol..

[53] Hoffmann M., Hofmann-Winkler H., Kruger N., Kempf A., Nehlmeier I., Graiche L., Arora P., Sidarovich A., Moldenhauer A.-S., Winkler M.S. (2021). SARS-CoV-2 variant B.1.617 is resistant to Bamlanivimab and evades antibodies induced by infection and vaccination. Cell Rep..

[54] Ashwaq O., Manickavasagam P., Haque S.M. (2021). V483A: An emerging mutation hotspot of SARS-CoV-2. Future Virol..

[55] Yadav P.D., Mohandas S., Shete A.M., Nyayanit D.A., Gupta N., Patil D.Y., Sapkal G.N., Potdar V., Kadam M., Kumar A. (2021). SARS CoV-2 variant B.1.617.1 is highly pathogenic in hamsters than B.1 variant. bioRxiv.

[56] Kim S., Lei Z., Dicker J., Cao Y., Zhang X.F., Im W. (2021). Differential interactions between human ACE2 and spike RBD of SARS-CoV-2 variants of concern. bioRxiv Prepr. Server Biol..

[57] Laffeber C., de Koning K., Kanaar R., Lebbink J.H.G. (2021). Experimental evidence for enhanced receptor binding by rapidly spreading SARS-CoV-2 variants. J. Mol. Biol..

[58] Shahhosseini N.G., Babuadze G., Wong G., Kobinger G.P. (2021). Mutation signatures and in silico docking of novel SARS-CoV-2 variants of concern. Microorganisms.

[59] Liu H., Wei P., Zhang Q., Chen Z., Aviszus K., Downing W., Peterson S., Reynoso L., Downey G.P., Frankel S.K. (2021). 501Y.V2 and 501Y.V3 variants of SARS-CoV-2 lose binding to Bamlanivimab in vitro. bioRxiv Prepr. Server Biol..

[60] Chakraborty S. (2021). Evolutionary and structural analysis elucidates mutations on SARS-CoV2 spike protein with altered human ACE2 binding affinity. Biochem. Biophys. Res. Commun..

[61] Bastolla U., Demetrius L. (2005). Stability constraints and protein evolution: The role of chain length, composition and disulfide bonds. Protein Eng. Des. Sel..

[62] Schwede T., Kopp J., Guex N., Peitsch M.C. (2003). SWISS-MODEL: An automated protein homology-modeling server. Nucleic Acids Res..

[63] Waterhouse A., Bertoni M., Bienert S., Studer G., Tauriello G., Gumienny R., Heer F.T., de Beer T.A.P., Rempfer C., Bordoli L. (2018). SWISS-MODEL: Homology modelling of protein structures and complexes. Nucleic Acids Res..

[64] Laskowski R.A., MacArthur M.W., Moss D.S., Thornton J.M. (1993). PROCHECK: A program to check the stereochemical quality of protein structures. J. Appl. Crystallogr..

[65] Yan Y., Zhang D., Zhou P., Li B., Huang S.Y. (2017). HDOCK: A web server for protein-protein and protein-DNA/RNA docking based on a hybrid strategy. Nucleic Acids Res..

[66] Yan Y., Tao H., He J., Huang S.Y. (2020). The HDOCK server for integrated protein–protein docking. Nat. Protoc..

[67] Laskowski R.A., Swindells M.B. (2011). LigPlot+: Multiple ligand-protein interaction diagrams for drug discovery. J. Chem. Inf. Model..

[68] Laskowski R.A., Jabłońska J., Pravda L., Vařeková R.S., Thornton J.M. (2018). PDBsum: Structural summaries of PDB entries. Protein Sci..

[69] Abraham M.J., Murtola T., Schulz R., Pall S., Smith J.C., Hess B., Lindahl E. (2015). Gromacs: High performance molecular simulations through multi-level parallelism from laptops to supercomputers. SoftwareX.

[70] Lindorff-Larsen K., Piana S., Palmo K., Maragakis P., Klepeis J.L., O’Dror R., Shaw D.E. (2010). Improved side-chain torsion potentials for the Amber ff99SB protein force field. Proteins Struct. Funct. Bioinform..

[71] Wang E., Sun H., Wang J., Wang Z., Liu H., Zhang J.Z.H., Hou T. (2019). End-point binding free energy calculation with MM/PBSA and MM/GBSA: Strategies and applications in drug design. Chem. Rev..

[72] Kumari R., Kumar R., Lynn A. (2014). G-mmpbsa-A GROMACS tool for high-throughput MM-PBSA calculations. J. Chem. Inf. Model..

